# Genomic Change, Retrotransposon Mobilization and Extensive Cytosine Methylation Alteration in *Brassica napus* Introgressions from Two Intertribal Hybridizations

**DOI:** 10.1371/journal.pone.0056346

**Published:** 2013-02-28

**Authors:** Xueli Zhang, Xianhong Ge, Yujiao Shao, Genlou Sun, Zaiyun Li

**Affiliations:** 1 National Key Laboratory of Crop Genetic Improvement, National Center of Crop Molecular Breeding Technology, National Center of Oil Crop Improvement (Wuhan), College of Plant Science and Technology, Huazhong Agricultural University, Wuhan, People’s Republic of China; 2 College of Chemistry and Life Science, Hubei University of Education, Wuhan, People’s Republic of China; 3 Department of Biology, Saint Mary’s University, Halifax, Canada; VU University Medical Center, The Netherlands

## Abstract

Hybridization and introgression represent important means for the transfer and/or *de novo* origination of traits and play an important role in facilitating speciation and plant breeding. Two sets of introgression lines in *Brassica napus* L. were previously established by its intertribal hybridizations with two wild species and long-term selection. In this study, the methods of amplified fragment length polymorphisms (AFLP), sequence-specific amplification polymorphism (SSAP) and methylation-sensitive amplified polymorphism (MSAP) were used to determine their genomic change, retrotransposon mobilization and cytosine methylation alteration in these lines. The genomic change revealed by the loss or gain of AFLP bands occurred for ∼10% of the total bands amplified in the two sets of introgressions, while no bands specific for wild species were detected. The new and absent SSAP bands appeared for 9 out of 11 retrotransposons analyzed, with low frequency of new bands and their total percentage of about 5% in both sets. MSAP analysis indicated that methylation changes were common in these lines (33.4–39.8%) and the hypermethylation was more frequent than hypomethylation. Our results suggested that certain extents of genetic and epigenetic alterations were induced by hybridization and alien DNA introgression. The cryptic mechanism of these changes and potential application of these lines in breeding were also discussed.

## Introduction

Interspecific hybridization is recognized as a major force in plant evolution by contributing to diversification and speciation [1], [2]. Hybridization has been reported to lead to speciation in three ways: allopolyploidy from the duplication of chromosome complement in a hybrid [3]; homoploid hybrid and recombinational speciation where new hybrid species share the same ploid as their parents [2]; and introgression from backcrossing of the hybrids to parents [4]. While homoploid hybrid speciation is generally regarded as rare, allopolyploidy is common and may be responsible for the origins of most plant species believed to be recent or ancient polyploids [5]. It has long been recognized that hybridization and introgression occur widely among natural plant populations [6]. Although introgressive hybridization does not necessarily lead to immediate hybrid speciation, it is an important mean for the transfer and/or *de novo* origination of traits related to ecological adaptations, therefore, plays an important role in facilitating speciation in changing niches [7].

The role of hybridization on plant genome was primarily thought as the “genomic shock” by McClintock [8], which leads to genome-wide relaxation of gene expression including transposon activation. The process is often accompanied with rapid and extensive genomic, and epigenomic changes which causes global gene expression alteration, including fragment gain and loss by chromosome rearrangement or activation of transposable element [9]–[11], extensive alteration of DNA cytosine methylation [12], [13], histone modification [14] and small RNA change [15]. These changes are considered as a stabilizing mechanism for the establishment of new species [14]–[17]. A key role of introgressive hybridization is possibly to generate extensive stochastic genomic and epigenomic variation that can be translated into phenotypic novelties and upon which natural selection may then act [18].

**Figure 1:**
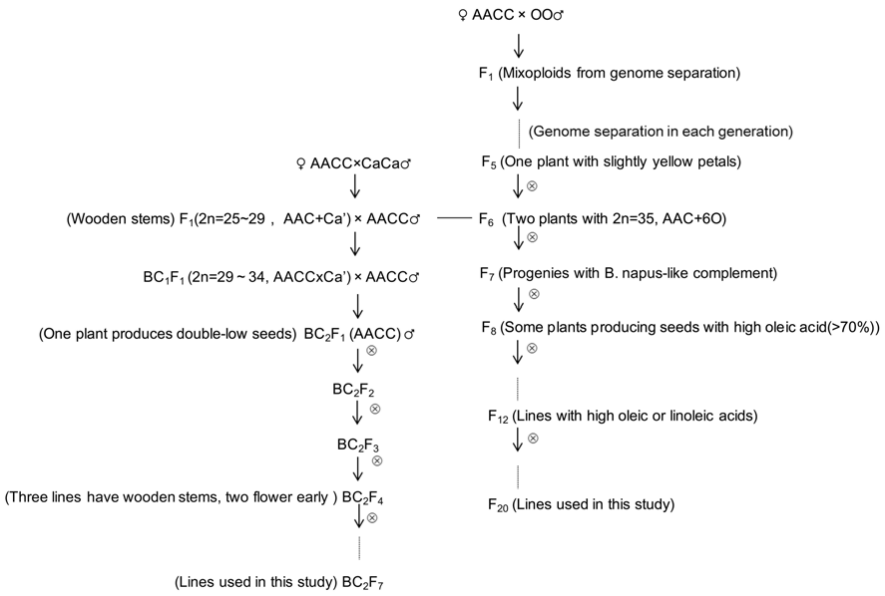
Pedigrees of *B. napus* introgression lines from two crosses with two wild species. AACC: *Brassica napus*. CaCa: *Capsella bursa-pastoris*. OO: *Orychophragmus violaceus*. Ca’: individual chromosome or chromosomal segments from *C. bursa-pastoris*. Cx: uncertain number of the chromosomes from C genome of *B. oleracea*. The short line between two crosses indicates the generation involving the hybrid or progeny with the partial *B. napus* complement and some alien chromosomes or segments from which the introgressions are derived.

Interspecific hybridization is also widely used to introgress alien genetic elements from wild species into crops to increase genetic variability or transfer desirable traits. In the crosses involving two distantly related species, hybrids with unexpected chromosomal and genomic compositions are often produced, which resulted from the early elimination of alien fragments or chromsomes of the male parent during the hybrid embryo development, genome rearrangements consecutive to ‘genomic shock’ [8], [19] and diploidization [20], [21]. Such hybrids have female-parent-type phenotypes and chromosome numbers but altered genomic compositions and have been reported in several plants, including coffee [22], rapeseed [21], [23]–[27], rice [28] and sunflower [20]. The intertribal hybrids between *Brassica napus* L. (2n = 38, AACC) and two other crucifers *Orychophragmus violaceus* (L.) O.E. Schulz (2n = 24, OO), *Capsella bursa-pastoris* (L.) Medic (2n = 4x = 32) produced by us turned out to be partial hybrids [25], [29], and stable introgression lines have been established by successive selections [30], [31] (Figure 1). *Orychophragmus violaceus* was suggested to have a phylogenetic position outside of the tribe *Brassiceae* [32], [33], and *C. bursa-pastoris* belongs to the tribe *Lepidieae*. These lines have the same karyotype as *B. napus* but with genomic change, and some of their phenotypes are different from *B. napus* by expressing some traits specific for wild species and novel characters. These introgressions have two obvious attributes: they are derivatives of partial sexual hybrids between very distantly related species, and are the products of long artificial selections, up to 20 generations. Comparison of their genetic and epigenetic components with *B. napus* parents should reveal the extent of the genomic restructuring of recipient *B. napus* genomes during the relatively long period of hybridization and introgression, and elucidate the general patterns of genetic and epigenetic alteration and stabilization. Our present results indicated that foreign DNA induced genetic recombination of recipient genomes accompanied with extensive DNA cytosine methylation changes, but the activation of retrotransposons occurred for restricted elements.

## Results

### Development of *B. napus* Introgressions from Two Intertribal Hybridizations

Two sets of introgression lines in *Brassica napus* L. (2n = 38, AACC) were developed from its intertribal crosses and used in this study (Figure 1). One set of six lines was derived from one partial intertribal hybrid between *B. napus* cv. Zhongyou 821 and *Capsella bursa-pastoris* (L.) Medic (2n = 4x = 32) [25] (designated as cross A). Zhongyou 821 was one elite cultivar with the largest acreage during 1980s–1990s in China, with the merit of high seed yield and good resistance to *Sclerotinia sclerotiorum*, and was used as the parents of many cultivars. But it had double-high seed quality (high content of erucic acid and glucosinolates). *C. bursa-pastoris* possessed high resistance to *S. sclerotiorum* which was probably related to its lignified or wooden stems, and early flowering, and the accession used in the cross was double-low. This hybrid showed some obvious characters of *C. bursa-pastoris* origin, such as the clustering stems, small deep-green and serrated leaves, the red color on stems and leaf veins, and particularly wooden stems. The hybrid was probably the result of the elimination of the chromosomes from *C. bursa-pastoris* with some chromosomal segments introgressed and also the elimination of some chromosomes from *B. napus*, for it had 2n = 29 in most somatic cells, likely with the complement 20A +9C [25]. The hybrid was backcrossed twice to Zhongyou 821 as pollen parent and the progenies were self-crossed for six generations and selected for double-low quality, wooden stems and early flowering (Figure 1). One BC_2_F_1_ plant produced selfed seeds with nearly double-low quality, and after three more generations, it gave rise to three BC_2_F_4_ lines which had wooden stems and also double-low quality. Two BC_2_F_4_ lines were found to flower about one month earlier than *B. napus* parent but with double-high quality. The other lines had double-low quality and some phenotypic variations. These lines were further selfed for several generations and selected for fertility, specific morphology and seed quality. Finally, the cytologically stable (2n = 38) and good seed-set lines were obtained, particularly the ones with wooden stems and double-low quality, and the ones which flowered about one month earlier than *B. napus* parent but had a longer flowering duration and also double-high quality (Table S1). The lines with wooden stems were found to have 10–20% higher content of lignin in their stems and the changed plant architecture with branches being tightly compressed to the main inflorescence, and also showed lodging resistance, owing to their wooden and rigid stems. The wooden stems also enhanced resistance to *S. sclerotiorum*, likely resulted from the altered plant architecture.

Another set of six lines was derived from one mixoploidy hybrid between *B. napus* cv. ‘Oro’ and *Orychophragmus violaceus* (designated as cross B) (Figure 1). The Oro was the first *B. napus* cultivar with low content of erucic acid in the world, which was selected from one local variety ‘Liho’ in Germany, and was introduced to China in 1974 and used in breeding programs as the donor for the genes controlling low erucic acid [34]. But Oro had high content of glucosinolates. *O. violaceus* with large purple flowers, an ornamental crucifer in China, was found to possess favorable fatty acid compositions (∼50% linoleic acid and ∼1% erucic acid) [35]. The mixoploidy hybrid with the separation of parental genomes during mitotic division was successively selected always for mixoploidy to F_5_ generation and a single plant with slight yellow petals obtained [30], [31] (Figure 1). This F_5_ plants showed the altered chromosome behavior and also produced F_6_ plants with variable chromosomal complements. Two F_6_ plants produced the further progenies with high percentage of oleic acid (∼70%, 10% higher than that of ‘Oro’), increased level of linoleic acid and widely variable levels of glucosinolate contents. These plants advanced to F_20_ had the karyotype of *B. napus* but with genomic changes including some fragments from *O. violaceus*. They also showed some phenotypic deviations from Oro, by expressing some traits from *O. violaceus*, such as basic clustering stems, serrated leaves, and also the novel white or slightly yellow flowers (Table S1).

Although the two sets of introgressions were derived from two different crosses, their formation experienced similar cytological processes with some introgression of alien genetic elements (Figure 1), which was probably responsible for the similar genetic and epigenetic changes detected below. In cross A, the progeny hybrids for the introgressions had the main chromosome complement (AAC) with individual chromosomes from male parent *C. bursa-pastoris*, and the *B. napus* karyotype was recovered by backcrossing to *B. napus* parent twice and stabilized by selfing several times. In cross B with *O. violaceus*, the somatic cells of F_1_ hybrid contained different chromosome numbers, and F_1_ hybrid produced mainly two types of plants: the hybrid and *B. napus*, due to separation of the parental genomes during mitotic and meiotic divisions of the hybrid cells (2n = 31, ACO) [29], [36]. The hybrid plants in F_2_ to F_4_ populations showed similar chromosome behavior as F_1_ hybrids. But one F_5_ plant exhibiting the phenotype of hybrid except for the light yellow petals showed the alteration of chromosome segregation and subsequently produced F_6_ progenies with different phenotypes and chromosome numbers [37]. The two progeny F_6_ plants (F_6_–8, 9, 2n = 35) for the lines used in this study likely have the chromosome complements (AAC+6O), and produced the progenies with *B. napus*-like complement plus some incorporated segments of *O. violaceus*. Then introgressions were selected and finalized by selfing for several generations (Figure 1). So the two sets of lines contained genetic materials from *B. napus* complement (AAC) and some alien chromosomes, which was reorganized and subsequently the *B. napus* karyotype was recovered during the process of backcrossing and selfing.

### Genomic Changes Revealed by AFLP Analysis in *B. napus* Introgressions

The genomic components of six BC_2_F_6_ lines derived from cross A and the six F_20_ lines derived from cross B were analyzed using AFLP and compared with crossing parents. Using 50 different primer pairs, 1596 and 1545 clear bands were averagely obtained for each line in cross A and cross B, respectively, but no bands specific for either *C. bursa-pastoris* or *O. violaceus* were detected in any lines. Three types of bands were scored in these lines: parental *B. napus* bands, lost bands from *B. napus* parents, and novel bands unobserved in *B. napus* parents. The limited differences existed in the number and frequency of each type of bands between lines (Table 1). Among the lines from cross A, 5.1–6.0% bands of *B. napus* were not detected in the progenies with the average of 5.4%, 5.4–6.4% bands were novel with the average of 5.9%. The average rate of loss (5.4%) was significantly lower than that of gain (5.9%). Among the lines from cross B, the percentages of absent and novel bands were 6.8–7.8%, 5.0–5.7%, respectively. The average percentage of loss (7.2%) was significantly higher than that of gain (5.4%). To compare the change between two crosses, the average rate of loss in cross A (5.4%) was significantly lower than that in cross B (7.2%) (P<0.01), but that of gain (5.9%) in the cross A was significantly higher than 5.4% in cross B (P<0.05). But the sum rate of gain and loss in cross A (11.3%) was lower than that in cross B (12.6%) (P<0.01) (Table 1). These introgressions largely maintained the genomic components of *B. napus* parent but altered at certain extents.

**Table 1:** The number and percentage of absent and novel AFLP bands in *B. napus* introgression lines.

	A-1	A-2	A-3	A-4	A-5	A-6	Mean	B-1	B-2	B-3	B-4	B-5	B-6	Mean
**Loss**	77	78	81	85	79	91	81.8	103	110	115	107	104	99	106.3
**(%)**	(5.1)	(5.1)	(5.3)	(5.6)	(5.2)	(6.0)	(5.4)^a^ **	(7.0)	(7.5)	(7.8)	(7.3)	(7.1)	(6.8)	(7.2)^a^ **
**Gain**	82	97	93	90	96	83	90.2	84	81	75	75	82	74	78.5
**(%)**	(5.4)	(6.4)	(6.1)	(5.9)	(6.3)	(5.5)	(5.9)^b^*	(5.7)	(5.5)	(5.1)	(5.1)	(5.6)	(5.0)	(5.4)^c^*
**Total**	159	175	174	175	175	174	172	187	191	190	182	186	173	184.8
**(%)**	(10.5)	(11.5)	(11.5)	(11.5)	(11.5)	(11.5)	(11.3)**	(12.7)	(13.0)	(12.9)	(12.4)	(12.7)	(11.8)	(12.6) **

*The average rate of gain in cross A (5.9%) was significantly higher than 5.4% in cross B (P<0.05).

** The average rates of loss (5.4% and 7.2%) and the total rates (11.3% and 12.6%) in two crosses were significantly different (P<0.01).

a–b There was a significant difference between the mean frequency of loss and gain in cross A (P<0.05).

a–c There was an extremely significant difference between the mean frequency of loss and gain in cross B (P<0.01).

### Retrotransposon Alterations in Introgressions Revealed by SSAP

**Figure 2:**
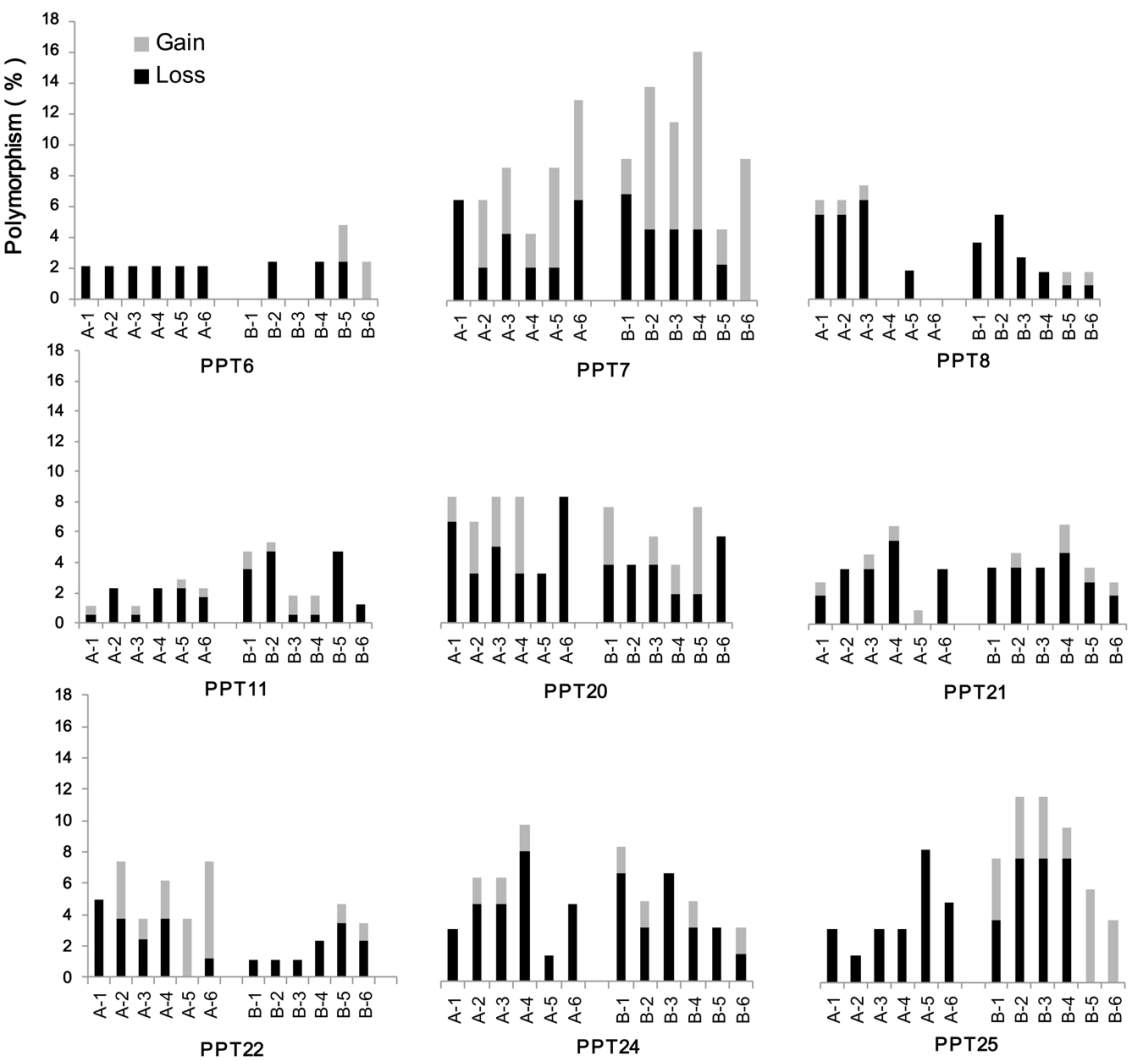
Genetic changes for SSAP bands in 12 introgressions from two crosses.

**Figure 3:**
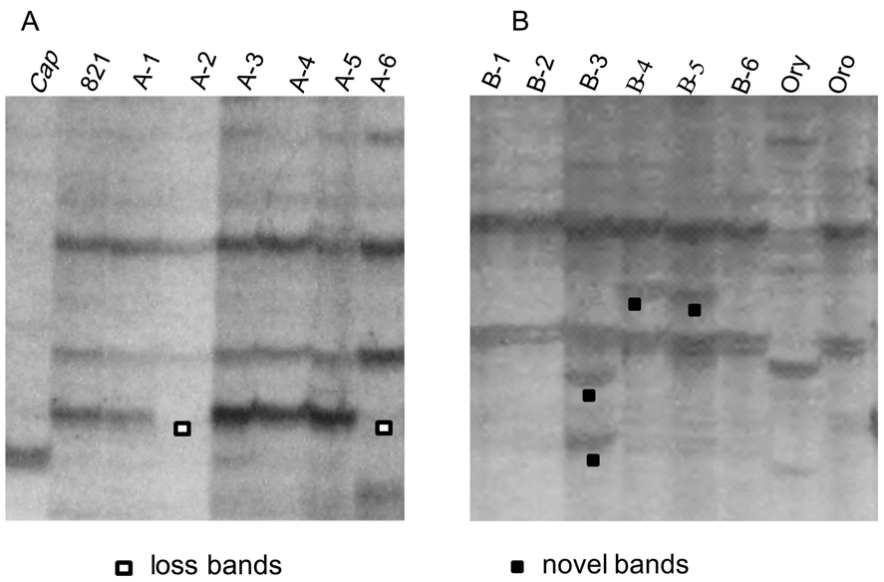
Examples of typical SSAP patterns detected in 12 introgression lines and their parents. The gel profiles in parts A and B are produced by primer combinations of *Bfa-A+PPT20*, *Bfa-A+PPT8*, respectively. In part A, *Cap* is for *C. bursa-pastoris* and 821 for *B. napus* ‘821’ and. In part B, *Ory* is for *O. violaceus* and Oro for *B. napus* ‘Oro’.

Retrotransposons are the most abundant and widespread class of plant transposable element, which can rapidly increase their copy number and greatly increase plant genome size by “copy-paste” replicative mode of transposition [38]. As one of retrotransponsons, LTR (long terminal repeat) retrotransposons were commonly used to detect the mobilization of transposable elements in hybrids/allopolyploids and under stress conditions [9], [39]–[42]. Herein, to explore the alterations of retrotransposons in the two sets of lines, 11 LTR (*Copia*) retrotransposons detected in *B. rapa* were investigated in these introgressions. SSAP polymorphisms of these retrotransposons were compared across these lines and parents, and the absent and new bands were scored (Figures 2, 3). No new and absent bands were found for PPT 12, 17. For the remaining 9 retrotransposons, the frequency of loss and gain bands was generally low, with average of 3.6% and 1.3% for cross A, and 3.2% and 2.0% for cross B, respectively, while more than 90% parental bands were maintained in the progenies (Table S2, Table 2; Figure 2). The overall frequency of absent bands (3.4%) was higher than that of the new bands (1.6%), and the highest frequencies of new bands occurred for PPT 7 at 3.9% in cross A and 6.8% in cross B (Table 2). Most of lines from two crosses had both absent and novel bands, some lines had only absent bands or novel bands. The line A-5 from cross A showed only novel bands for PPT 21, 22, and the line B-6 from cross B had only novel bands for PPT 6, 7. Different lines from the same cross displayed variations in pattern and frequency of changes for each retrotransposon, except all lines from cross A with the same frequency of only absent bands for PPT 6(Table S2).

**Table 2:** The percentage of absent and novel SSAP bands in *B. napus* introgression lines.

	Loss%		Gain%		Total%	
	Cross A	Cross B	Cross A	Cross B	Cross A	Cross B
**PPT 6**	2.2^cd^	1.3^c^	0.0^c^	0.8^cd^	2.2^d^	2.1^c^
**PPT 7**	3.9^abcd^	3.8^ab^	3.9^a^	6.8^a^	7.8^a^	10.6^a^
**PPT 8**	3.4^abcd^	2.7^abc^	0.5^c^	0.3^d^	3.9^bcd^	3.0^c^
**PPT 11**	1.6^d^	2.6^abc^	0.4^c^	0.7^cd^	2.0^d^	3.3^c^
**PPT 20**	5.5^a^	3.9^ab^	2.5^ab^	2.5^bc^	8.0^a^	6.4^b^
**PPT 21**	2.8^bcd^	3.2^abc^	0.6^c^	0.7^cd^	3.4^cd^	3.9^bc^
**PPT 22**	2.7^cd^	1.9^bc^	2.9^a^	0.4^d#^	5.6^abc^	2.3^c##^
**PPT 24**	5.2^ab^	4.8^a^	0.9^bc^	1.3^cd^	6.1^ab^	6.1^b^
**PPT 25**	4.6^abc^	5.1^a^	0.0^c^	4.3^b^	4.6^bcd^	9.4^a#^
**Means**	3.6	3.2	1.3**	2.0*	4.9	5.2

** There was an extremely significant difference between the mean frequency of loss and gain in cross A.

* There was a significant difference between the mean frequency of loss and gain in cross B.

a–d The values in each column sharing the same letters show no significant differences (P<0.05).

# ## There was a significant or an extremely significant difference in the total frequencies between the cross A and B.

For each retrotransposon, the lines from two crosses also gave certain differences in pattern and frequency, at extreme case, all lines from cross A had only absent bands for PPT 6, 25 (Table S2; Figure 2). However, although different frequencies for loss, gain and their sum within each retrotransposon were given by the lines from cross A and B, the significant differences appeared only between their percentages of gain (2.9% for cross A/0.4% for cross B) and the total (5.6%/2.3%) for PPT 22, and between the total (4.6%/9.4%) for PPT 25 (Table 2).

For the differences in the frequencies of changes across 9 different retrotransposons within each line from two crosses (Table 2, Table S2; Figure 2), the number and rate of loss ranged from 0 to 8, 0% to 9.3%, respectively; the number and rate of gain ranged from 0 to 5, 0% to 11.4%, respectively; the number and rate of total changes were from 0 to 9, 0% to 15.9%. Within each line, there existed some differences in the number and rate of loss, gain and the total change among different retrotransposons, while the patterns of variations shown by different lines varied for most of retrotransposons studied. For the total rate of three changes among 12 lines from two crosses, significant differences appeared only for loss (1.6–4.3%), but not for gain (0.5–2.3%) and their total (3.9–6.2%). The rates of loss in B-2 (4.3%), A-1 (4.0%), A-6 (3.9%) and B-1 (3.9%) were comparable and were significantly higher than that in B-6 (1.6%) (Table 3).

**Table 3:** The total percentages of absent and novel SSAP bands in each introgression line.

		Loss%	Gain%	Total%
**Cross A**	**A-1**	4.0^a^	0.5_**_	4.5
	**A-2**	3.4^ab^	1.6_*_	5.0
	**A-3**	3.8^a^	1.5_*_	5.3
	**A-4**	3.6^ab^	1.4	5.0
	**A-5**	2.6^ab^	1.3	3.9
	**A-6**	3.9^a^	1.5	5.4
**Cross B**	**B-1**	3.9^a^	1.6_*_	5.5
	**B-2**	4.3^a^	1.9	6.2
	**B-3**	3.7^ab^	1.6	5.3
	**B-4**	3.4^ab^	2.3	5.7
	**B-5**	2.5^ab^	2.3	4.8
	**B-6**	1.6^b^	2.3	3.9
**Mean**		3.4	1.6**	5.0

*** There was a significant difference between the frequency of loss and gain within each line (P<0.05 or 0.01).

a–b The values in each column sharing the same letters show no significant differences (P<0.01).

### Extensive DNA Methylation Changes in Introgressions

**Figure 4:**
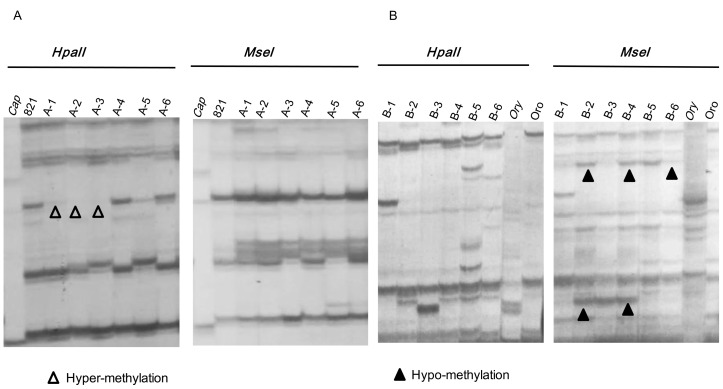
Examples of typical MSAP patterns detected in 12 introgression lines and their parents. The gel profiles in parts A and B were produced by primer combinations of *EcorI*-ACT+*H/M*-TAG and *EcorI*-ACT+*H/M-TTC,* respectively. The denotations for the parents are the same as in Figure 3.

To reveal epigenetic alterations in these introgressions, MSAP analyses that targeted randomly sampled genomic loci were performed. By using 40 pairs of randomly selected primers, an average of 1063 and 1017 clear and reproducible bands was given by each line in cross A and B, respectively. According to the model of alteration in the introgressions, the MSAP loci could be grouped into 15 patterns in comparison with those bands in *B. napus* parents. These three patterns were excluded for further analysis: ++/++, ++/− and −/++, because it was difficult to decide whether there were methylated loci (++/++) or the alterations resulting from the methylation changes or sequence variation (restriction enzyme cutting site change) (++/− and −/++). The remaining 12 patterns were classified into four types (Table 4, Table S3). Type A included two patterns (A1, A2) representing the additive bands from *B. napus* parents, and the site number of A2 pattern was more than double of the number of A1 pattern in the two crosses. The additive bands accounted for 62.3% and 64.6% of the total bands observed in cross A and B, respectively. Among the four patterns in Type B that showed hypermethylation by gain of methylated loci at one or both CCGG sites (Figure 4), twice as many bands occurred for B2 pattern as other three types in both crosses, which presented 23.3% and 20.3% of the total loci in cross A and B, respectively, and the frequency for cross A was significantly higher than that for cross B. Other four patterns in Type C indicated hypomethylation by release of methylated loci at one or both CCGG sites (Figure 4), and C1 pattern had much fewer number than the other three which showed similar numbers. The frequencies of bands for hypomethylation were 14.0% in cross A and 14.3% in cross B, which was much lower than those for hypermethylation. Type D included two other methylation patterns which occurred at low rates, 0.3% and 0.8% in cross A and B, respectively. Overall, methylation changes were common in these lines (33.4–39.8%) and the hypermethylation was more frequent than hypomethylation. For each pattern of methylation, variation in frequencies among lines from the same or different crosses was very limited, as their genomic change and retrotransposon alteration did.

**Table 4:** Cytosine methylation patterns in *B. napus* introgression lines revealed by MSAP analysis.

Type	Class	Patterns				Number and frequency of sites	
		*B. napus*		Introgressions		cross A	cross B
		H	M	H	M		
**Additive**	**A1**	+	–	+	–		
	**A2**	–	+	–	+		
	**Total**					270.0(62.3%)	263.8(64.6%)
**Hyper**	**B1**	+	+	+	–		
	**B2**	+	+	–	+		
	**B3**	+	–				
	**B4**	–	+				
	**Total**					101.3(23.3%)	82.8(20.3%**)
**Hypo**	**C1**	+	–	+	+		
	**C2**	–	+	+	+		
	**C3**			+	–		
	**C4**			–	+		
	**Total**					61.0(14.0%)	58.3(14.3%)
**Others**	**D1**	+	–	–	+		
	**D2**	–	+	+	–		
	**Total**					1.5(0.3%)	3.2(0.8%)

** There was an extremely significant difference between the mean frequency of hypermethylation in cross A and B.

The relationships among genetic and epigenetic changes revealed by AFLP, SSAP and MSAP were different in the introgressions from two crosses (Table 5). In cross A, AFLP fragments were positively correlated with MSAP and SSAP ones, in spite of low coefficient, but MSAP and SSAP fragments were negatively correlated. In cross B, three type fragments were positively correlated, significantly between AFLP and MSAP (r = 0.83, P<0.05), and between MSAP and SSAP (r = 0.71, P<0.05).

**Table 5:** Percentage and correlation of genetic and epigenetic changes in *B. napus* introgression lines from two crosses.

	Introgression lines from Cross A									Introgression lines from Cross B								
	A-1	A-2	A-3	A-4	A-5	A-6	rAM	rAS	rMS	B-1	B-2	B-3	B-4	B-5	B-6	rAM	rAS	rMS
**AFLP**	10.49	11.54	11.48	11.54	11.54	11.48	0.51			12.76	13.04	12.97	12.42	12.70	11.81	0.83*		
**MSAP**	35.28	34.81	36.92	39.50	39.60	39.77		0.23		35.29	35.89	36.19	35.97	35.32	33.42		0.46	
**SSAP**	3.33	3.90	3.90	3.67	2.76	3.67			−0.36	3.99	4.58	3.40	3.76	3.64	2.58			0.71*

Note: rAM, rAS and rMS represent coefficient of correlation between the genetic changes revealed by AFLP and MSAP, AFLP and SSAP, and MSAP and SSAP, respectively.

## Discussion

In this study, *B. napus* introgression lines derived from its intertribal partial hybrids with two other species (*C. bursa-pastoris*,*O. violaceus*) showed genomic component restructuring (DNA sequences and retrotransposons) and methylation status change at certain extents. The absence of alien DNA fragments possibly resulted from the limited primer number used, or the few sequences introgressed, for these lines expressed some traits of paternal donors. Similarly, genome scan of hybridizing (*Helianthus annuus* and *H. debilis*) sunflowers reveals asymmetric patterns of introgression and small islands of genomic differentiation [43]. These changes were the consequences of genomic rearrangements and introgressions following hybridization, backcrossing and self-pollinating (Figure 1), although the alien chromosomes and chromosomal segments were gradually lost as the generation advanced. These changes induced by wide hybridization and subsequent introgression were finally stabilized by directional selections for fertility, specific characters and stable karyotype.

### Genetic Changes in *B. napus* Introgressions from Wide Hybridization

*B. napus* which belongs to the tribe *Brassiceae* is phylogenetically distant from *C. bursa-pastoris* and *O. violaceus*, for *C. bursa-pastoris* is included in the tribe *Lepidieae* and *O. violaceus* was suggested to excluded from the tribe *Brassiceae* [32], [33], because an independent mesotetraploid WGD (whole-genome duplication) in *Orychophragmus* was revealed by comparative chromosome painting [32]. Such wide relationships might cause the chromosome elimination and the production of partial hybrids from their intertribal hybridizations [25], [30], [31]. As only individual chromosomes or chromosomal segments from *C. bursa-pastoris* were detected in mitotic and meiotic cells of partial hybrids with *B. napus* [25], possibility of meiotic recombination between *B. napus* and alien chromosomes was quite low. Different timing and spatial separation of parental chromosomes in sexual hybrids between *B. napus* and *O. violaceus* should prevent the homoeologous pairing and recombination between the chromosomes of two species [29], [37]. So genome restructuring accompanied by the translocation of alien segments following hybridization and backcrossing resulted in the genomic deviations of these introgressions from *B. napus* parents. The similar extents of genetic and epigenetic changes in the introgressions from two crosses might be attributable to their similar developmental pathways. Furthermore, the similar genetic component of these introgressions suggests some restrictions on the genome structure. Previous analysis of recombination in the homoploid hybrid sunflower (*Helianthus anomalus*) showed that newly synthesized hybrids converged on a linkage pattern similar to that of wild hybrids within five generations [44].

The changes in genomic constitution, DNA methylation and gene expression were shown to accompany with the allopolyploidization process of resynthesized *B. napus* [45]–[48]. *De novo* genetic alterations associated with retrotransposon activation, genomic rearrangements and phenotypic variation were detected in the *B. napus* introgressions with alien chromosomal segments from natural *B. rapa* [48]. In stable rice introgression lines derived from intergeneric hybridization between rice (*Oryza sativa* L.) and a wild relative (*Zizania latifolia* Griseb.) followed by successive selfing, up to 30% genomic changes were caused by introgression of a small amount (<0.1%) of foreign DNA [18]. It was suggested that the changes happened at very early stage by cryptic pathway other than by conventional or unorthodox meiotic recombination of homeoalleles between rice and *Zizania* [18]. The more genomic change in rice introgressions is probably attributable to the diploidy nature of rice genome, while the tetraploid *B. napus* genome is more tolerable for genomic shock.

The genetic changes in these introgressions might result from the earlier incorporation of alien genetic elements into *B. napus*, from the activation of transposable elements or DNA methylation, as suggested in rice introgression lines [18]. The other reasons for the production of novel and deleted bands (Table 1) might be chromosomal rearrangements through intergenomic translocations/transpositions [49] and homoeologous pairing [50], [51], because *Brassica* A and C genomes are considered to be derived from a common ancestral genome and thus partially homologous [52], [53]. Widespread chromosomal rearrangements caused by homoeologous recombination had been detected in *B. napus* [50], [51] and have consequences for allelic and phenotypic diversity [46], [49], [54], 55. Accordingly, the overall frequencies of bands loss and gain are similar (Table 1), although it needs to confirm which loci changes resulted from meiotic recombination. The production of the novel and deleted bands might also be related to rapid sequence elimination, as has been reported in synthetic hybrids and allopolyploids of *Brassica*, triticale and *Aegilops–Triticum* [45], [56], [57]. The similar frequency of genetic changes in two crosses (Table 1) suggests that the genetic changes are less related to the original parents and more to consecutive selection.

### Retrotransposon Mobilization and Methylation Changes in Introgressions

Transposable elements (TEs) represent a major component of plant genomes which are usually transcriptionally silent. Interspecific hybridization has been proposed to induce bursts of transposition attributable to the interaction of merged genomes, as first proposed by McClintock [8]. However, recent studies focusing on different allopolyploid species failed to provide evidence for transposition bursts [9], [58], [59]. Our study on 11 retrotransposons (*Copica*) in these *B. napus* introgressions revealed some changes at low frequency, even no changes were detected for PPT 12 and PPT 17 (Table 2, Table S2; Figure 2). The changes of SSAP bands in introgression lines varied among different retrotransposons and the lost bands were more frequent than new bands (Table S2; Figure 2). Fragment loss as a general trend has been reported in most polyploids and is usually more frequent than putative TE transpositions (reviewed in [60]). The loss and gain of SSAP bands were caused not only by retrotransposons activation but also by other processes, such as indels at TE insertion sites or rearrangements encompassing TEs insertion sites [41]. Therefore, the sequence changes revealed by comparisons of the SSAP profiles of the parental species and the introgression lines were partly caused by TE activations, so a transposition burst did not occur for the retrotransposons studied here.

The cytosine methylation plays an important role in epigenetic gene regulation in vertebrates and higher plants [61]. In contrast to animals, where methylated cytosine residues are primarily observed within the symmetrical CpG dinucleotide, plants display cytosine methylation in any DNA context, including symmetric CG and CHG (where H = A, T or C) and asymmetric CHH [62]. Our results showed that methylation pattern alterations occurred in introgression lines at high rates (37.7% in cross A, 35.4% in cross B) (Table 4, Table S3). The proportion of methylation changes appeared to be variable among the plant hybrid/allopolyploid systems analyzed to date using MSAP. In cotton, MSAP fragment additivity was observed in nearly all cases [63]. In resynthesized allotetraploid *Arabidopsis suecica*, methylation changes represented 8.3% of the fragments [64]. Higher proportion of parent fragments changes here indicated that the extensive DNA methylation changes were probably induced by wide hybridization as found in *Spartina*, in which 30% parental methylation patterns were altered in the hybrids and the allopolyploid [65].

In plants, three DNA methyltransferases: MET1, CMT3, and DRM2 and four bifunctional 5-methylcytosine glycosylases: Repressor of silencing 1 (ROS1), Demeter (DME), DME-like 2 (DML2), and DML3 are found responding for DNA methylation and demethylation [66]. The methylation loci detected here, CpG methylation are maintained after DNA replication and during cell division by *METHYLTRANSFERASE 1* (*MET1*) that catalyzes preferentially hemimethylated substrates [67], [68]. The methylation changes found here might be related to the hybridization-associated enzymatic machinery changes. Moreover, recent studies suggested that both siRNAs and long noncoding RNAs were involved in *de novo* DNA methylation [69]. The changes of DNA methylation here might also result from small RNA expression changes induced by hybridization and allopolyploidization [15]. Transposable elements and other repetitive sequences are the primary targets of DNA methylation [70]. Accordingly, it was found that alterations in hypermethylation surpassed those in hypomethylation in two crosses. We proposed that presumably some of transposable elements were activated after hybridization but were then silenced, as indicated by high frequency of hypermethylation detected here.

### Genetic and Epigenetic Changes and Phenotypic Variation

Genetic and epigenetic changes revealed here resulted in extensive phenotypic variation of introgressions (Table S1). Interestingly, some traits exhibited by individuals are desirable to increase adaptation and diversity in *B. napus*, and these lines could serve as new germplasm for plant breeding. For instance, the introgression lines from the cross with *C. bursa-pastoris* exhibited strong resistance to lodging and stem rot, the most serious disease in China. The plant architecture with compressed branches and rigid stems was suitable for high-density planting and mechanical harvesting. This also shows that the hybridization and introgression play an important role in producing novel and key germplasm for breeding programs.

## Materials and Methods

### Plant Material

Two sets of six introgression lines each in *Brassica napus* L. (2n = 38, AACC) were developed and used in this study: A1–A6 from the intertribal cross between *B. napus* cv. Zhongyou 821 and *Capsella bursa-pastoris* (L.) Medic (2n = 4x = 32), and B1–B6 from the intertribal cross between *B. napus* cv. Oro and *Orychophragmus violaceus* (L.) O.E. Schulz (2n = 24, OO) (Figure 1). The selfed seeds of introgression lines and parental lines were sown in the field of Huazhong Agriculture University in Wuhan in early October, 2010. About 4 weeks later, the leaves of young plants were collected for DNA extraction.

### Amplified Fragment Length Polymorphism (AFLP) Analysis

Genomic DNA was extracted using the cetyl-trimethyl-ammonium-bromide method [71]. The AFLP analysis was performed according to the standard AFLP procedure [72]. In total, 50 pairs of randomly selective primers were used for amplifications with one technical replicate, and only clear and reproducible bands were scored.

### Sequence-specific Amplification Polymorphism (SSAP) Analysis

The sequence-specific amplification polymorphism (SSAP) technique [73] is a method based on retrotransposon marker that combines the high resolution of AFLP with the specificity of an oligonucleotide primer anchored on the terminal sequences of a retrotransposon, usually in the long terminal repeat (LTR). The primers for eleven retrotransposons PPT 6, PPT 7, PPT 8, PPT 11, PPT 12, PPT 17, PPT 20, PPT 21, PPT 22, PPT 24 identified in *Brassica rapa* were employed to detect DNA polymorphisms with one technical replicate based on the location of the retrotransposons relative to adjacent restriction endonuclease, as described by Zou [48] (Table S4).

### Methylation-Sensitive Amplification Polymorphism (MSAP) Analysis

MSAP analysis was performed according to the methylation-sensitive amplified fragment length polymorphism (MSAP) protocol [47], [74]. MSAP involved the use of isoschizomers, *HpaII* and *MspI* that recognize the same CCGG sites, but have different sensitivity to the methylation states of the cytosine. The *EcoRI* remains unchanged. *Hpa*II is inactive if one or both cytosines are fully methylated (both strands methylated), but cleaves hemi-methylated sequences (only a single DNA strand is methylated) or no methylation sequences; whereas, *Msp*I digests inner methylation of double-stranded DNA or no methylation. Based on the comparison of presence (+) or absence (−) of a band from the combination of *EcoR* I/*Hpa* II (H) and *EcoR* I/*Msp* I (M) digested genomic DNA, the methylation status of the specific loci could be decided. Namely,++,+–, –+and – – represents CCGG/GGCC or C ^5m^CGG/GGCC, ^5m^CCGG/GGCC, C ^5m^CGG/GGC^5m^ C and ^5m^CCGG/GGCC^5m^, respectively. Forty pairs of randomly selective primers were employed to detect the DNA methylation alterations pattern with one technical replicate, and only clear and reproducible bands were scored.

### Fatty Acid, Oil Content, and Glucosinolate Analysis

Seed oil was extracted and its composition was analyzed using gas chromatography machine (HP 6890, Germany). The fatty acid and glucosinolate contents were detected using nearinfrared reflectance spectroscopy (NIRS) (Vector 22/N, Bruker, Germany).

### Statistical Analysis

Two-by-two chi-squared contingency tests were used to test significance of genetic changes between introgressions. The *t* tests were performed to compare the Pearson correlations among these changes.

## Supporting Information

Table S1 **Phenotypes and seed quality of** ***B. napus*** **introgression lines with some characters from wild species.**
(DOCX)

Table S2 **The genomic variation in** ***B. napus*** **introgression lines based on SSAP analysis.**
(DOCX)

Table S3 **Alteration of cytosine methylation patterns in** ***B. napus*** **introgression lines revealed by MSAP analysis.**
(DOC)

Table S4 **The primer sequence for sequence-specific amplification polymorphism (SSAP) related markers.**
(DOCX)
