# Supplementary material for: Genomic Change, Retrotransposon Mobilization and Extensive Cytosine Methylation Alteration in Brassica napus Introgressions from Two Intertribal Hybridizations
Source: PLoS One. 2013 Feb 28;8(2):e56346. doi: 10.1371/journal.pone.0056346 (PMC3585313; doi:10.1371/journal.pone.0056346)
Supplement: Table S1 — Phenotypes and seed quality of B. napus introgression lines with some characters from wild species. (DOCX) [file pone.0056346.s001.docx]

Table S1. Phenotypes and seed quality of *B. napus* introgression lines with some characters from wild species

|  | Introgression lines from Cross A | | | | | | | | Introgression lines from Cross B | | | | | | | |
| --- | --- | --- | --- | --- | --- | --- | --- | --- | --- | --- | --- | --- | --- | --- | --- | --- |
|  | *C.bursa-pastoris* | 821 | A-1 | A-2 | A-3 | A-4 | A-5 | A-6 | O. violaceus | Oro | B-1 | B-2 | B-3 | B-4 | B-5 | B-6 |
| **Serrated leaves & clustering stems** | - | - | - | - | - | - | - | - | + | - | - | - | - | + | - | - |
| **Wooden stems** | + | - | - | + | + | - | - | - | - | - | - | - | - | - | - | - |
| **Early flowering** | + | - | - | - | - | + | + | + | - | - | - | - | - | - | - | - |
| **Double-Low seed quality^a^** | + | - | + | + | - | - | - | - | - | - | - | - | - | - | - | - |
| **>70% oleic acid** | - | - | - | - | - | - | - | - | - | - | + | + | + | + | - | - |

^a^The components of glucosinolates(μmol/g oil free meal) were lower than 30μmol/g oil free meal, the content of erucic acid were lower than 1%.
