# Supplementary material for: Genomic Change, Retrotransposon Mobilization and Extensive Cytosine Methylation Alteration in Brassica napus Introgressions from Two Intertribal Hybridizations
Source: PLoS One. 2013 Feb 28;8(2):e56346. doi: 10.1371/journal.pone.0056346 (PMC3585313; doi:10.1371/journal.pone.0056346)
Supplement: Table S2 — The genomic variation in B. napus introgression lines based on SSAP analysis. (DOCX) [file pone.0056346.s002.docx]

**Table S2.** The genomic variation in *B. napus* introgression lines based on SSAP analysis

|  | | **Introgression lines from cross A** | | | | | | | **Introgression lines from cross B** | | | | | | |
| --- | --- | --- | --- | --- | --- | --- | --- | --- | --- | --- | --- | --- | --- | --- | --- |
|  | | **821** | **A-1** | **A-2** | **A-3** | **A-4** | **A-5** | **A-6** | **Oro** | **B-1** | **B-2** | **B-3** | **B-4** | **B-5** | **B-6** |
| PPT6 | **Loss** |  | 1 | 1 | 1 | 1 | 1 | 1 |  | 0 | 1 | 0 | 1 | 1 | 0 |
|  | **%** |  | (2.2) | (2.2) | (2.2) | (2.2) | (2.2) | (2.2) |  | (0.0) | (2.5) | (0.0) | (2.5) | (2.5) | (0.0) |
|  | **Gain** |  | 0 | 0 | 0 | 0 | 0 | 0 |  | 0 | 0 | 0 | 0 | 1 | 1 |
|  | **%** |  | (0.0) | (0.0) | (0.0) | (0.0) | (0.0) | (0.0) |  | (0.0) | (0.0) | (0.0) | (0.0) | (2.5) | (2.5) |
|  | **Total** |  | 1 | 1 | 1 | 1 | 1 | 1 |  | 0 | 1 | 0 | 1 | 2 | 1 |
|  | **%** |  | (2.2) | (2.2) | (2.2) | (2.2) | (2.2) | (2.2) |  | (0.0) | (2.5) | (0.0) | (2.5) | (5.0) | (5.0) |
|  | **Total** | 45 | 44 | 44 | 44 | 44 | 44 | 44 | 40 | 40 | 39 | 40 | 39 | 40 | 41 |
| PPT7 | **Loss** |  | 3 | 1 | 2 | 1 | 1 | 3 |  | 3 | 2 | 2 | 2 | 1 | 0 |
|  | **%** |  | (6.4) | (2.1) | (4.3) | (2.1) | (2.1) | (6.4) |  | (6.8) | (4.5) | (4.5) | (4.5) | (2.3) | (0.0) |
|  | **Gain** |  | 0 | 2 | 2 | 1 | 3 | 3 |  | 1 | 4 | 3 | 5 | 1 | 4 |
|  | **%** |  | (0.0) | (4.3) | (4.3) | (2.1) | (6.4) | (6.4) |  | (2.3) | (9.1) | (6.8) | (11.4) | (2.3) | (9.1) |
|  | **Total** |  | 3 | 3 | 4 | 2 | 4 | 6 |  | 4 | 6 | 5 | 7 | 2 | 4 |
|  | **%** |  | (6.4) | (6.4) | (8.5) | (4.3) | (8.5) | (12.8) |  | (9.1) | (13.6) | (11.4) | (15.9) | (4.5) | (9.1) |
|  | **Total** | 47 | 44 | 48 | 47 | 47 | 49 | 47 | 44 | 42 | 46 | 45 | 47 | 44 | 48 |
| PPT8 | **Loss** |  | 6 | 6 | 7 | 0 | 2 | 0 |  | 4 | 6 | 3 | 2 | 1 | 1 |
|  | **%** |  | (5.8) | (5.8) | (6.7) | (0) | (1.9) | (0.0) |  | (3.8) | (5.7) | (2.9) | (1.9) | (1.0) | (1.0) |
|  | **Gain** |  | 1 | 1 | 1 | 0 | 0 | 0 |  | 0 | 0 | 0 | 0 | 1 | 1 |
|  | **%** |  | (1.0) | (1.0) | (1.0) | (0.0) | (0.0) | (0.0) |  | (0.0) | (0.0) | (0.0) | (0.0) | (1.0) | (1.0) |
|  | **Total** |  | 7 | 7 | 8 | 0 | 2 | 0 |  | 4 | 6 | 3 | 2 | 2 | 2 |
|  | **%** |  | (6.7) | (6.7) | (7.7) | (0.0) | (1.9) | (0.0) |  | (3.8) | (5.7) | (2.9) | (1.9) | (1.9) | (1.9) |
|  | **Total** | 104 | 98 | 98 | 97 | 104 | 102 | 104 | 105 | 101 | 99 | 102 | 103 | 105 | 105 |
| PPT11 | **Loss** |  | 1 | 4 | 1 | 4 | 4 | 3 |  | 6 | 8 | 1 | 1 | 8 | 2 |
|  | **%** |  | (0.6) | (2.3) | (0.6) | (2.3) | (2.3) | (1.7) |  | (3.6) | (4.7) | (0.6) | (0.6) | (4.7) | (1.2) |
|  | **Gain** |  | 1 | 0 | 1 | 0 | 1 | 1 |  | 2 | 1 | 2 | 2 | 0 | 0 |
|  | **%** |  | (0.6) | (0.0) | (0.6) | (0.0) | (0.6) | (0.6) |  | (1.2) | (0.6) | (1.2) | (1.2) | (0.0) | (0.0) |
|  | **Total** |  | 2 | 4 | 2 | 4 | 5 | 4 |  | 8 | 9 | 3 | 3 | 8 | 2 |
|  | **%** |  | (1.2) | (2.3) | (1.2) | (2.3) | (2.9) | (2.3) |  | (4.7) | (5.3) | (1.8) | (1.8) | (4.7) | (1.2) |
|  | **Total** | 172 | 172 | 168 | 172 | 168 | 169 | 170 | 169 | 165 | 162 | 170 | 170 | 161 | 167 |
| PPT12 | **Loss** |  | 0 | 0 | 0 | 0 | 0 | 0 |  | 0 | 0 | 0 | 0 | 0 | 0 |
|  | **%** |  | (0.0) | (0.0) | (0.0) | (0.0) | (0.0) | (0.0) |  | (0.0) | (0.0) | (0.0) | (0.0) | (0.0) | (0.0) |
|  | **Gain** |  | 0 | 0 | 0 | 0 | 0 | 0 |  | 0 | 0 | 0 | 0 | 0 | 0 |
|  | **%** |  | (0.0) | (0.0) | (0.0) | (0.0) | (0.0) | (0.0) |  | (0.0) | (0.0) | (0.0) | (0.0) | (0.0) | (0.0) |
|  | **Total** |  | 0 | 0 | 0 | 0 | 0 | 0 |  | 0 | 0 | 0 | 0 | 0 | 0 |
|  | **%** |  | (0.0) | (0.0) | (0.0) | (0.0) | (0.0) | (0.0) |  | (0.0) | (0.0) | (0.0) | (0.0) | (0.0) | (0.0) |
|  | **Total** | 67 | 67 | 67 | 67 | 67 | 67 | 67 | 72 | 72 | 72 | 72 | 72 | 72 | 72 |
| PPT17 | **Loss** |  | 0 | 0 | 0 | 0 | 0 | 0 |  | 0 | 0 | 0 | 0 | 0 | 0 |
|  | **%** |  | (0.0) | (0.0) | (0.0) | (0.0) | (0.0) | (0.0) |  | (0.0) | (0.0) | (0.0) | (0.0) | (0.0) | (0.0) |
|  | **Gain** |  | 0 | 0 | 0 | 0 | 0 | 0 |  | 0 | 0 | 0 | 0 | 0 | 0 |
|  | **%** |  | (0.0) | (0.0) | (0.0) | (0.0) | (0.0) | (0.0) |  | (0.0) | (0.0) | (0.0) | (0.0) | (0.0) | (0.0) |
|  | **Total** |  | 0 | 0 | 0 | 0 | 0 | 0 |  | 0 | 0 | 0 | 0 | 0 | 0 |
|  | **%** |  | (0.0) | (0.0) | (0.0) | (0.0) | (0.0) | (0.0) |  | (0.0) | (0.0) | (0.0) | (0.0) | (0.0) | (0.0) |
|  | **Total** | 76 | 76 | 76 | 76 | 76 | 76 | 76 | 76 | 76 | 76 | 76 | 76 | 76 | 76 |
| PPT20 | **Loss** |  | 4 | 2 | 3 | 2 | 2 | 5 |  | 2 | 2 | 2 | 1 | 1 | 3 |
|  | **%** |  | (7.4) | (3.7) | (5.6) | (3.7) | (3.7) | (9.3) |  | (4.3) | (4.3) | (4.3) | (2.1) | (2.1) | (6.4) |
|  | **Gain** |  | 1 | 2 | 2 | 3 | 0 | 0 |  | 2 | 0 | 1 | 1 | 3 | 0 |
|  | **%** |  | (1.9) | (3.7) | (3.7) | (5.6) | (0.0) | (0.0) |  | (4.3) | (0.0) | (2.1) | (2.1) | (6.4) | (0.0) |
|  | **Total** |  | 5 | 4 | 5 | 5 | 2 | 5 |  | 4 | 2 | 3 | 2 | 4 | 3 |
|  | **%** |  | (9.3) | (7.4) | (9.3) | (9.3) | (3.7) | (9.3) |  | (8.5) | (4.3) | (6.4) | (4.3) | (8.5) | (6.4) |
|  | **Total** | 54 | 51 | 54 | 53 | 55 | 52 | 49 | 47 | 47 | 45 | 46 | 47 | 49 | 44 |
| PPT21 | **Loss** |  | 2 | 4 | 4 | 6 | 0 | 4 |  | 4 | 4 | 4 | 5 | 3 | 2 |
|  | **%** |  | (1.7) | (3.4) | (3.4) | (5.1) | (0.0) | (3.4) |  | (3.5) | (3.5) | (3.5) | (4.3) | (2.6) | (1.7) |
|  | **Gain** |  | 1 | 0 | 1 | 1 | 1 | 0 |  | 0 | 1 | 0 | 2 | 1 | 1 |
|  | **%** |  | (0.9) | (0.0) | (0.9) | (0.9) | (0.9) | (0.0) |  | (0.0) | (0.9) | (0.0) | (1.7) | (0.9) | (0.9) |
|  | **Total** |  | 3 | 4 | 5 | 7 | 1 | 4 |  | 4 | 5 | 4 | 7 | 4 | 3 |
|  | **%** |  | (2.6) | (3.4) | (4.3) | (6.0) | (0.8) | (3.4) |  | (3.5) | (4.3) | (3.5) | (6.1) | (3.5) | (2.6) |
|  | **Total** | 117 | 116 | 113 | 114 | 112 | 118 | 113 | 115 | 111 | 112 | 111 | 112 | 113 | 114 |
| PPT22 | **Loss** |  | 4 | 3 | 2 | 3 | 0 | 1 |  | 1 | 1 | 1 | 2 | 3 | 2 |
|  | **%** |  | (4.9) | (3.7) | (2.5) | (3.7) | (0.0) | (1.2) |  | (1.2) | (1.2) | (1.2) | (2.3) | (3.5) | (2.3) |
|  | **Gain** |  | 0 | 3 | 1 | 2 | 3 | 5 |  | 0 | 0 | 0 | 0 | 1 | 1 |
|  | **%** |  | (0.0) | (3.7) | (1.2) | (2.5) | (3.7) | (6.2) |  | (0.0) | (0.0) | (0.0) | (0.0) | (1.2) | (1.2) |
|  | **Total** |  | 4 | 6 | 3 | 5 | 3 | 6 |  | 1 | 1 | 1 | 2 | 4 | 3 |
|  | **%** |  | (4.9) | (7.4) | (3.7) | (6.3) | (3.7) | (7.4) |  | (1.2) | (1.2) | (1.2) | (1.2) | (2.3) | (3.4) |
|  | **Total** | 81 | 77 | 81 | 80 | 80 | 84 | 85 | 86 | 85 | 85 | 85 | 84 | 84 | 85 |
| PPT24 | **Loss** |  | 2 | 3 | 3 | 5 | 1 | 3 |  | 4 | 2 | 4 | 2 | 2 | 1 |
|  | **%** |  | (3.7) | (5.6) | (5.6) | (9.3) | (1.9) | (5.6) |  | (7.7) | (3.8) | (7.7) | (3.8) | (3.8) | (1.9) |
|  | **Gain** |  | 0 | 1 | 1 | 1 | 0 | 0 |  | 1 | 1 | 0 | 1 | 0 | 1 |
|  | **%** |  | (0.0) | (1.9) | (1.9) | (1.9) | (0.0) | (0.0) |  | (1.9) | (1.9) | (0.0) | (1.9) | (0.0) | (1.9) |
|  | **Total** |  | 2 | 4 | 4 | 6 | 1 | 3 |  | 5 | 3 | 4 | 3 | 2 | 2 |
|  | **%** |  | 3.7 | 7.5 | 7.5 | 11.2 | 1.9 | 5.6 |  | 9.6 | 5.7 | 7.7 | 5.7 | 3.8 | 3.8 |
|  | **Total** | 54 | 52 | 52 | 52 | 50 | 53 | 51 | 52 | 49 | 51 | 48 | 51 | 50 | 52 |
| PPT25 | **Loss** |  | 2 | 1 | 2 | 2 | 5 | 3 |  | 2 | 4 | 4 | 4 | 0 | 0 |
|  | **%** |  | (3.7) | (1.9) | (3.7) | (3.7) | (9.3) | (5.6) |  | (4.3) | (8.7) | (8.7) | (8.7) | (0.0) | (0.0) |
|  | **Gain** |  | 0 | 0 | 0 | 0 | 0 | 0 |  | 2 | 2 | 2 | 1 | 3 | 2 |
|  | **%** |  | (0.0.) | (0.0) | (0.0) | (0.0) | (0.0) | (0.0) |  | (4.3) | (4.3) | (4.3) | (2.2) | (6.5) | (4.3) |
|  | **Total** |  | 2 | 1 | 2 | 2 | 5 | 3 |  | 4 | 6 | 6 | 5 | 3 | 2 |
|  | **%** |  | (3.7) | (1.9) | (3.7) | (3.7) | (9.3) | (5.6) |  | (8.7) | (13.0) | (13.0) | (10.9) | (6.5) | (4.3) |
|  | **Total** | 54 | 52 | 51 | 52 | 52 | 49 | 51 | 46 | 46 | 44 | 44 | 43 | 49 | 48 |
