# Supplementary material for: Genomic Change, Retrotransposon Mobilization and Extensive Cytosine Methylation Alteration in Brassica napus Introgressions from Two Intertribal Hybridizations
Source: PLoS One. 2013 Feb 28;8(2):e56346. doi: 10.1371/journal.pone.0056346 (PMC3585313; doi:10.1371/journal.pone.0056346)
Supplement: Table S3 — Alteration of cytosine methylation patterns in B. napus introgression lines revealed by MSAP analysis. (DOC) [file pone.0056346.s003.doc]

**Table S3.** Alteration of cytosine methylation patterns in *B. napus* introgression lines revealed by MSAP analysis

| **Type** | **Class** | **Pattern** | | | | **Number and frequency of sites** | | | | | | | | | | | | | |
| --- | --- | --- | --- | --- | --- | --- | --- | --- | --- | --- | --- | --- | --- | --- | --- | --- | --- | --- | --- |
|  |  | ***B. napus*** | | **Introgressions** | | **Introgression lines from cross A** | | | | | | | **Introgression lines from cross B** | | | | | | |
|  |  | **H** | **M** | **H** | **M** | **A-1** | **A-2** | **A-3** | **A-4** | **A-5** | **A-6** | **Mean** | **B-1** | **B-2** | **B-3** | **B-4** | **B-5** | **B-6** | **Mean** |
| **Additive** | **A1** | + | - | + | - | 85 | 87 | 82 | 83 | 81 | 82 |  | 75 | 75 | 78 | 76 | 71 | 74 |  |
|  | **A2** | - | + | - | + | 192 | 192 | 188 | 182 | 189 | 183 |  | 189 | 193 | 183 | 191 | 189 | 189 |  |
|  | **Total** |  |  |  |  | 277 | 279 | 270 | 265 | 270 | 265 | 271.0 | 264 | 268 | 261 | 267 | 260 | 263 | 263.8 |
|  | **%** |  |  |  |  | 64.7 | 65.2 | 63.1 | 60.5 | 60.4 | 60.2 | 62.3 | 64.7 | 64.1 | 63.8 | 64.0 | 64.7 | 66.6 | 64.6 |
| **Hyper** | **B1** | + | + | + | - | 18 | 20 | 19 | 21 | 21 | 17 |  | 15 | 20 | 16 | 18 | 14 | 14 |  |
|  | **B2** | + | + | - | + | 40 | 40 | 43 | 42 | 49 | 47 |  | 35 | 38 | 37 | 39 | 38 | 38 |  |
|  | **B3** | + | - |  |  | 19 | 17 | 20 | 20 | 20 | 22 |  | 11 | 13 | 12 | 11 | 15 | 13 |  |
|  | **B4** | - | + |  |  | 18 | 18 | 18 | 20 | 16 | 23 |  | 14 | 14 | 26 | 14 | 15 | 17 |  |
|  | **Total** |  |  |  |  | 95 | 95 | 100 | 103 | 106 | 109 | 101.3 | 75 | 85 | 91 | 82 | 82 | 82 | 82.8 |
|  | **%** |  |  |  |  | 22.2 | 22.2 | 23.4 | 23.5 | 23.7 | 24.7 | 23.3 | 18.4 | 20.3 | 22.2 | 19.7 | 20.4 | 20.8 | 20.3 |
| **Hypo** | **C1** | + | - | + | + | 0 | 2 | 1 | 4 | 5 | 4 |  | 5 | 3 | 5 | 4 | 6 | 6 |  |
|  | **C2** | - | + | + | + | 17 | 12 | 12 | 13 | 16 | 19 |  | 9 | 8 | 4 | 10 | 9 | 6 |  |
|  | **C3** |  |  | + | - | 22 | 22 | 23 | 26 | 26 | 22 |  | 23 | 22 | 24 | 21 | 17 | 14 |  |
|  | **C4** |  |  | - | + | 16 | 17 | 20 | 22 | 24 | 21 |  | 27 | 28 | 23 | 29 | 26 | 21 |  |
|  | **Total** |  |  |  |  | 55 | 52 | 56 | 65 | 71 | 66 | 61.0 | 64 | 61 | 56 | 64 | 58 | 47 | 58.3 |
|  | **%** |  |  |  |  | 12.9 | 12.4 | 13.1 | 14.8 | 15.9 | 15.0 | 14.0 | 15.7 | 14.6 | 13.7 | 15.3 | 14.4 | 11.9 | 14.3 |
| **Others** | **D1** | + | - | - | + | 1 | 0 | 1 | 1 | 0 | 0 |  | 3 | 4 | 1 | 4 | 2 | 2 |  |
|  | **D2** | - | + | + | - | 0 | 1 | 1 | 4 | 0 | 0 |  | 2 | 0 | 0 | 0 | 0 | 1 |  |
|  | **Total** |  |  |  |  | 1 | 1 | 2 | 5 | 0 | 0 | 1.5 | 5 | 4 | 1 | 4 | 2 | 3 | 3.2 |
|  | **%** |  |  |  |  | 0.2 | 0.2 | 0.5 | 1.1 | 0 | 0 | 0.3 | 1.2 | 1.0 | 0.2 | 1.0 | 0.5 | 0.8 | 0.8 |
