# Supplementary material for: Genomic Change, Retrotransposon Mobilization and Extensive Cytosine Methylation Alteration in Brassica napus Introgressions from Two Intertribal Hybridizations
Source: PLoS One. 2013 Feb 28;8(2):e56346. doi: 10.1371/journal.pone.0056346 (PMC3585313; doi:10.1371/journal.pone.0056346)
Supplement: Table S4 — The primer sequence for sequence-specific amplification polymorphism (SSAP) related markers. (DOCX) [file pone.0056346.s004.docx]

**Table S4. The primer sequence for sequence-speciﬁc ampliﬁcation polymorphism (SSAP) related markers**

| primers | Primer Resource (BAC Name) | Primer Sequence (5’-3’) |
| --- | --- | --- |
| PPT6 | LTR (AC189212) | ATTCGACTGAGGGGGGGG |
| PPT7 | LTR (AC155335) | ACTCTCCAACTTGAGGGGGAG |
| PPT8 | LTR (AC189254) | TCCTCCATCTTGAGGGGGAG |
| PPT11 | LTR (AC189187) | GTTTGCAGTTTAAGGGAGGGAT |
| PPT12 | LTR (AC189224) | TCAAGCTTAGGCGGGAGAA |
| PPT17 | LTR(AC189369) | GGTCCATCTTGAGGGGGTG |
| PPT20 | LTR(AC189373) | GTCCATCTTGAGGGGGCA |
| PPT21 | LTR(AC189374) | CATCAGTTTGCGGGGGGA |
| PPT22 | LTR(AC189386) | AAGTCGAGATATGATTAAGGAGGAG |
| PPT24 | LTR(AC189415) | TCTTTCCAAATTGAGGGGGAG |
| PPT25 | LTR(AC189470) | CAAGTTCAAGCTTAAAGGGGAGA |
